# Correction: Basit et al. Seed Priming with Spermine Mitigates Chromium Stress in Rice by Modifying the Ion Homeostasis, Cellular Ultrastructure and Phytohormones Balance. *Antioxidants* 2022, *11*, 1704

**DOI:** 10.3390/antiox14030312

**Published:** 2025-03-05

**Authors:** Farwa Basit, Javaid Akhter Bhat, Zaid Ulhassan, Muhammad Noman, Biying Zhao, Weijun Zhou, Prashant Kaushik, Ajaz Ahmad, Parvaiz Ahmad, Yajing Guan

**Affiliations:** 1 Institute of Crop Science, Zhejiang Key Laboratory of Crop Germplasm, Zhejiang University, Hangzhou 310058, China; 2 Hainan Research Institute, Zhejiang University, Sanya 572025, China; 3 International Genome Center, Jiangsu University, Zhenjiang 212013, China; javid.akhter69@gmail.com (J.A.B.);; 4 Independent Researcher, 46022 Valencia, Spain; 5 Department of Clinical Pharmacy, College of Pharmacy, King Saud University, Riyadh 11451, Saudi Arabia; 6 Department of Botany, GDC, Pulwama 192301, Jammu and Kashmir 192301, India

In the original publication [1], there was an error regarding the affiliation for author Prashant Kaushik. Specifically, affiliation number 4 (Instituto de Conservación y Mejora de la Agrodiversidad Valenciana, Universitat Politècnica de València, 46022 Valencia, Spain) has been removed from the affiliation list as per the institution’s request. Dr. Prashant Kaushik will be listed as an Independent Researcher. The fourth affiliation of Prashant Kaushik should be as follows:

Independent Researcher, 46022 Valencia, Spain

The authors state that the scientific conclusions are unaffected. This correction was approved by the Academic Editor. The original publication has also been updated.
