# Supplementary material for: Seed Priming with Spermine Mitigates Chromium Stress in Rice by Modifying the Ion Homeostasis, Cellular Ultrastructure and Phytohormones Balance
Source: Antioxidants (Basel). 2022 Aug 30;11(9):1704. doi: 10.3390/antiox11091704 (PMC9495668; doi:10.3390/antiox11091704)
Supplement: Supplementary file 1 [file antioxidants-11-01704-s001.zip › antioxidants-1831260-supplementary.pdf]

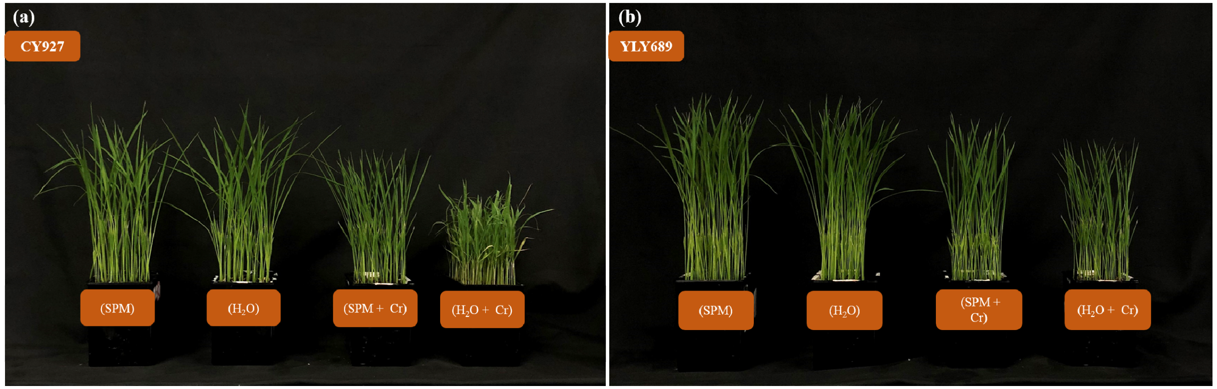

Figure S1. Effect of seed primed with SPM on phenotypic changes of two different rice cultivars under Cr toxicity. (a) Cr-induced phenotypical changes on the CY927 cultivar and mitigation effect of SPM under Cr stress. (b) Cr-induced phenotypical changes on the YLY689 cultivar and mitigation effect of SPM under Cr exposure.

Table S1. Primers information

Sr. #	Primer name	Accession No.	sequence
1	PR1-F	AU163470	ATGCTTCGTGCCTCCACTAC
2	PR1-R		GGCGAGTAGTTGCAGGTGAT
3	PR2-F	AY323485	ATTGAAGCAGGGAGTCGCAT
4	PR2-R		CGTGTGCTTGGTGCTGTATG
5	NPR1-F	DQ450947	AGCCCTTGACTCTGACGATG
6	NPR1-R		CAATGTTCGACGGCGTAGTG

Table S2. Effects of seed priming with Spm on net photosynthetic rate (Pn), transpiration rate (Tr), stomatal conductance (gs), intercellular concentration of CO_2 (Ci), and photochemical efficiency of PS II (Fv/Fm) under chromium (Cr) stress

**Genotypes**	**Treatments**	**Pn**	**Tr**	**Gs**	**Ci**	**Fv/Fm**
CY927	CK	7.43±0.06^{b}	4.25±0.13^{b}	0.25±0.00^{a}	466.44±1.86^{c}	0.62±0.02^{a}
	Spm	7.49±0.05^{b}	4.33±0.03^{b}	0.25±0.00^{a}	472.88±3.85^{bc}	0.63±0.02^{a}
	Cr	3.19±0.02^{f}	2.26±0.03^{f}	0.17±0.00^{d}	236.27±1.37^{g}	0.23±0.02^{e}
	Spm+Cr	5.43±0.05^{d}	2.81±0.05^{d}	0.19±0.00^{c}	274.99±4.44^{e}	0.35±0.01^{c}
YLY689	CK	7.90±0.01^{a}	4.64±0.01^{a}	0.25±0.00^{a}	479.09±3.96^{a}	0.63±0.02^{a}
	Spm	7.94±0.06^{a}	4.68±0.03^{a}	0.26±0.00^{a}	483.82±0.23^{a}	0.65±0.02^{a}
	Cr	4.30±0.08^{e}	2.64±0.01^{e}	0.19±0.00^{c}	267.77±1.44^{f}	0.31±0.01^{d}
	Spm+Cr	6.03b±0.07^{c}	3.57±0.02^{c}	0.21±0.00^{b}	316.35±3.17^{d}	0.41±0.02^{b}

The same letters within a column designate there was no significant difference at a 95% probability level at the $p < 0.05$ level, correspondingly.

Table S3. Effects of seed priming with Spm on Cr, micro as well as macronutrients uptake in shoots of both rice cultivars (CY927, YLY689) under chromium (Cr) stress

**Genotypes**	**Treatments**	**Na^{+}**	**Mg^{2+}**	**P**	**K^{+}**	**Ca^{2+}**	**Mn^{2+}**	**Fe^{2+}**	**Cu^{2+}**	**Zn^{2+}**	**Cr^{6+}**
		mg/g	mg/g	mg/g	mg/g	mg/g	mg/g	mg/g	mg/g	mg/g	mg/g
		Shoots									
CY-927	CK	$2.82±0.12^{cd}$	$3.57±0.30^{b}$	$10.23±0.24^{b}$	$34.05±2.11^{b}$	$3.76±0.22^{b}$	$1.49±0.06^{b}$	$0.21±0.09^{b}$	$0.03±0.01^{b}$	$0.09±0.03^{c}$	-
	SPM	$2.90±0.20^{c}$	$3.64±0.28^{b}$	$10.55±0.07^{b}$	$35.92±2.21^{b}$	$3.83±0.23^{b}$	$1.54±0.05^{b}$	$0.21±0.05^{b}$	$0.03±0.01^{b}$	$0.09±0.01^{c}$	-
	Cr	$2.69±0.02^{d}$	$2.18±0.13^{e}$	$6.83±0.1.27^{f}$	$22.85±1.67^{e}$	$2.25±0.17^{f}$	$0.31±0.05^{f}$	$0.05±0.04^{f}$	$0.01±0.00^{d}$	$0.05±0.01^{e}$	$1.93±0.04^{a}$
	SPM+Cr	$1.59±0.05^{f}$	$2.92±0.06^{c}$	$8.65±0.13^{d}$	$28.02±1.09^{d}$	$2.96±0.14^{d}$	$0.90±0.08^{d}$	$0.15±0.01^{e}$	$0.02±0.01^{c}$	$0.07±0.03^{d}$	$1.42±0.03^{c}$
YLY689	CK	$3.95±0.10^{a}$	$4.92±0.42^{a}$	$14.50±0.27^{a}$	$39.80±3.68^{a}$	$4.53±0.11^{a}$	$2.57±0.10^{a}$	$0.38±0.01^{a}$	$0.04±0.00^{a}$	$0.26±0.02^{a}$	-
	SPM	$4.01±0.34^{a}$	$5.17±0.23^{a}$	$15.68±0.65^{a}$	$41.67±1.53^{a}$	$4.61±0.07^{a}$	$2.66±0.06^{a}$	$0.42±0.04^{a}$	$0.04±0.00^{a}$	$0.27±0.01^{a}$	-
	Cr	$3.05±0.11^{bc}$	$2.49±0.51^{d}$	$7.56±0.55^{e}$	$28.22±2.53^{d}$	$2.50±0.12^{e}$	$0.53±0.11^{e}$	$0.17±0.02^{de}$	$0.02c±0.00^{c}$	$0.09c±0.01^{c}$	$1.73±0.03^{b}$
	SPM+Cr	$1.68±0.04^{e}$	$3.61±0.05^{b}$	$9.75±0.31^{c}$	$32.97±1.15^{c}$	$3.75±0.16^{c}$	$1.24±0.18^{c}$	$0.25±0.03^{c}$	$0.03±0.00^{b}$	$0.20b±0.02^{b}$	$1.46±0.03^{d}$

The same letters within a column designate there was no significant difference at a 95% probability level at the $p < 0.05$ level, correspondingly.

Table S4. Effects of seed priming with Spm on Cr, micro as well as macronutrients uptake in roots of both rice cultivars (CY927, YLY689) under chromium (Cr) stress

**Genotypes**	**Treatments**	**Na^{+}**	**Mg^{2+}**	**P**	**K^{+}**	**Ca^{2+}**	**Mn^{2+}**	**Fe^{2+}**	**Cu^{2+}**	**Zn^{2+}**	**Cr^{6+}**
		mg/g	mg/g	mg/g	mg/g	mg/g	mg/g	mg/g	mg/g	mg/g	mg/g
		Roots									
CY-927	CK	4.61±0.25^{c}	2.24±0.23^{ef}	5.51±0.23^{c}	12.50±0.22^{b}	3.34±0.33^{b}	0.39±0.02^{a}	5.26±0.09^{b}	0.18±0.01^{b}	0.10a±0.01^{b}	-
	SPM	4.53±0.08^{cd}	2.17±0.17^{f}	5.47±0.15^{c}	12.58±0.29^{b}	3.54±0.26^{b}	0.39±0.05^{a}	5.67±1.37^{b}	0.18±0.01^{b}	0.11a±0.02^{b}	-
	Cr	3.65±0.08^{e}	2.88±0.46^{c}	4.08±0.32^{f}	5.48±0.17^{f}	1.81±0.08^{f}	0.07±0.02^{e}	2.88±0.26^{f}	0.09±0.01^{e}	0.06b±0.01^{e}	2.65a±0.39^{b}
	SPM+Cr	3.09±0.28^{f}	2.59±0.12^{d}	4.64±0.21^{e}	8.89±0.24^{d}	2.41±0.31^{d}	0.21±0.06^{c}	3.64±0.13^{d}	0.14±0.01^{d}	0.08ab±0.02^{d}	0.50b±0.09^{c}
YLY689	CK	7.97±0.54^{a}	2.29±0.13^{e}	8.36±0.07^{a}	15.65±1.49^{a}	4.39±0.34^{a}	0.41±0.07^{a}	6.88±0.35^{a}	0.21±0.06^{a}	0.13a±0.03^{a}	-
	SPM	8.24±0.45^{a}	2.33±0.99^{e}	8.67±0.14^{a}	15.98±3.11^{a}	4.57±0.18^{a}	0.42±0.02^{a}	6.63±0.45^{a}	0.22±0.02^{a}	0.13a±0.05^{a}	-
	Cr	6.21±0.16^{b}	4.72±0.07^{a}	5.26±0.32^{d}	6.41±1.54^{e}	2.03±0.17^{e}	0.08±0.01^{de}	3.36±0.11^{e}	0.09±0.04^{e}	0.06c±0.04^{e}	3.31a±0.53^{a}
	SPM+Cr	4.46±1.02^{d}	4.17±0.24^{b}	6.90±0.25^{b}	9.47±2.07^{cd}	2.65±0.10^{c}	0.29±0.03^{b}	4.56±0.27^{c}	0.16±0.03^{c}	0.09b±0.02^{cd}	0.33b±0.51^{d}

The same letters within a column designate there was no significant difference at a 95% probability level at the $p < 0.05$ level, correspondingly.
